# Unmet Medication Needs and the Association with Mortality in Older Adults: Equality-Oriented Monitoring toward Universal Health Coverage

**DOI:** 10.14336/AD.2024.0626

**Published:** 2025-08-06

**Authors:** Minmin Wang, Yikai Feng, Yanan Luo, Yinzi Jin, Minghui Ren, Zhi-Jie Zheng

**Affiliations:** ^1^Department of Global Health, School of Public Health, Peking University, Beijing, China; ^2^Institute for Global Health and Development, Peking University, Beijing, China

**Keywords:** Unmet needs, universal health coverage, aging population, mortality

## Abstract

With population aging becoming a global trend, the unmet medication needs of older individuals are steadily increasing, potentially leading to a decline in quality of life and increased mortality. To identify unmet medication needs and inequality, and the association with health outcomes. A total of 69,443 participants in 31 countries from five international cohorts of aging were included. We measured the unmet medication needs level across sociodemographic strata. We developed an equality-oriented health care service coverage index (ESCI) and explored its relation to all-cause mortality in older adults over 55 years of age. Unmet medication needs in older adults with chronic conditions reached 41.84%. The highest unmet needs were observed in older age groups and participants with multimorbidity. The ESCI was further constructed by covering both the unmet needs level and inequality. An inverse association was observed between the ESCI and all-cause mortality in older adults (β=-16.81, *P*=0.047) as well as mortality rate owing to noncommunicable diseases (β=-17.58, *P*=0.041). The ESCI was inversely associated with mortality in older adults. This index could serve as a process evaluation indicator in assessing the progress toward UHC and healthy aging.

## INTRODUCTION

With population aging becoming a global trend, the unmet health care needs of older individuals are steadily increasing, potentially leading to a decline in quality of life and increased mortality, imposing an immense burden on individuals, health systems, and society [1]. Unmet health care needs are typically defined as the presence of health care needs for which people do not or cannot receive quality health care, although there is no globally agreed-upon definition [2-4]. Owing to the higher prevalence of chronic diseases and functional limitations among older adults, there is an increased demand for health care services in the context of population aging and a strong demand for diverse health care services especially medication and treatment for chronic diseases [5, 6]. This has led to an increase in the number of older people who have unmet medication needs, hindered the progress toward universal health coverage (UHC) (www.who.int/publications/i/item/9789240080379), and would have a disproportionate effect on health outcomes [7, 8].

UHC is the central target of health-related Sustainable Development Goals (SDGs) and is measured as the coverage of essential health services (SDG 3.8.1) and financial risk protection (SDG 3.8.2) [9]. However, neither metric measures unmet health care needs because only people who have received care are included. In 2023, the 76th World Health Assembly adopted a resolution requesting the World Health Organization (WHO) to review the importance and feasibility of using unmet needs for health care services as an additional indicator to monitor UHC nationally and globally. Older adults are especially vulnerable in receiving timely, quality, and affordable health care services. Nevertheless, current measures for monitoring progress toward UHC have not adequately captured older adults. Measuring unmet health care needs in vulnerable older adults could contribute to efforts to “leave no one behind” and to fortify global monitoring of UHC.

However, the current measurement of unmet medication needs in older individuals involves challenges regarding methodology and data availability [10]. First, current household-based surveys of unmet needs in older adults merely cover certain countries, with substantial variation across surveys in definitions and measurements of unmet needs [8, 11-13]. Second, it is crucial to evaluate their unmet needs while considering a range of socio demographic characteristics, and national averages should be presented alongside results of inequality monitoring [14]. Lastly, but most importantly, adverse health outcomes owing to unmet medication needs later in life have been largely understudied.

To address these research gaps, we established a set of indicators of unmet needs, integrated into an equality-oriented service coverage index (ESCI) to measure the unmet medication needs of older adults with chronic conditions based on five large, comparative cohort studies, including 69,443 older adults across 31 countries in North America, Europe, and Asia. We then investigated the association between unmet medication needs and mortality in older adults. This work will enrich the monitoring framework of progress toward UHC and healthy aging agenda.

## MATERIALS AND METHODS

### Data sources

Data were obtained from five international cohorts of aging: Health and Retirement Study (HRS), Survey of Health, Aging and Retirement in Europe (SHARE), China Health and Retirement Longitudinal Study (CHARLS), Mexican Health and Aging Study (MHAS), and Korean Longitudinal Study of Aging (KLoSA). The five surveys used here were designed to provide comparable results for health studies among older people across 31 countries, including Austria, Belgium, Bulgaria, China, Croatia, Cyprus, Czech Republic, Denmark, Estonia, Finland, France, Germany, Greece, Hungary, Israel, Italy, Latvia, Lithuania, Luxembourg, Malta, Mexico, the Netherlands, Poland, Republic of Korea, Romania, Slovakia, Slovenia, Spain, Sweden, Switzerland, and the United States. In this study, we included the survey data collected in 2018 for all five cohorts to evaluate the country-level unmet needs for the older population. We also obtained country-level indicators from World Bank Open Data and the WHO Global Health Observatory. Detailed information of the selected variables was listed in Supplemental Table 1.

### Definitions and measurements

*Unmet treatment needs:* In this study, the need for certain disease treatment services was defined as whether participants were previously diagnosed with certain chronic diseases. Details of the questions used to determine each participant’s need for medication in the HRS, SHARE, CHARLS, MHAS, and KLoSA surveys were listed in Supplementary Table 2.

Whether participants’ needs had been met was defined according to each participant’s response regarding whether they received medication or treatment related to their chronic condition(s). We defined participants with an unmet need for a chronic condition with which they had been diagnosed as those who did not receive any medications or treatments for the chronic condition. Details of the questions and coding forms used to define whether participants’ needs had been met in the HRS, SHARE, CHARLS, MHAS, and KLoSA surveys are listed in Supplementary Table 3.

The indicator for unmet medication needs level was defined as the proportion of participants who did not receive all medication or treatment that they needed among those with at least one common chronic disease (hypertension, diabetes, lung disease, and heart disease).

$$\mathrm{Unmet medication need level}=\frac{\mathrm{Number of participants did not receive targeted medication}}{\mathrm{Number of participants reporting being diagnosed with disease}}\times 100\%$$

*Inequality index and equality-oriented service coverage index:* A relative inequality index was calculated and an average value across the six dimensions (sex, residence, age group, income quintiles, education level, and multimorbidity) was defined as the inequality index.

$$\mathrm{Inequality across dimension}(i)=\left|\log\;(\frac{\mathrm{Lowest Unmet Need across subgroups in dimension}(i)}{\mathrm{Largest Unmet Need across subgroups in dimension}(i)})\right|$$

$$\mathrm{Inequality Index}=\frac{1}{n}\sum ({\mathrm{Inequality}}_{\mathrm{dimensions})}$$

**Figure 1.**
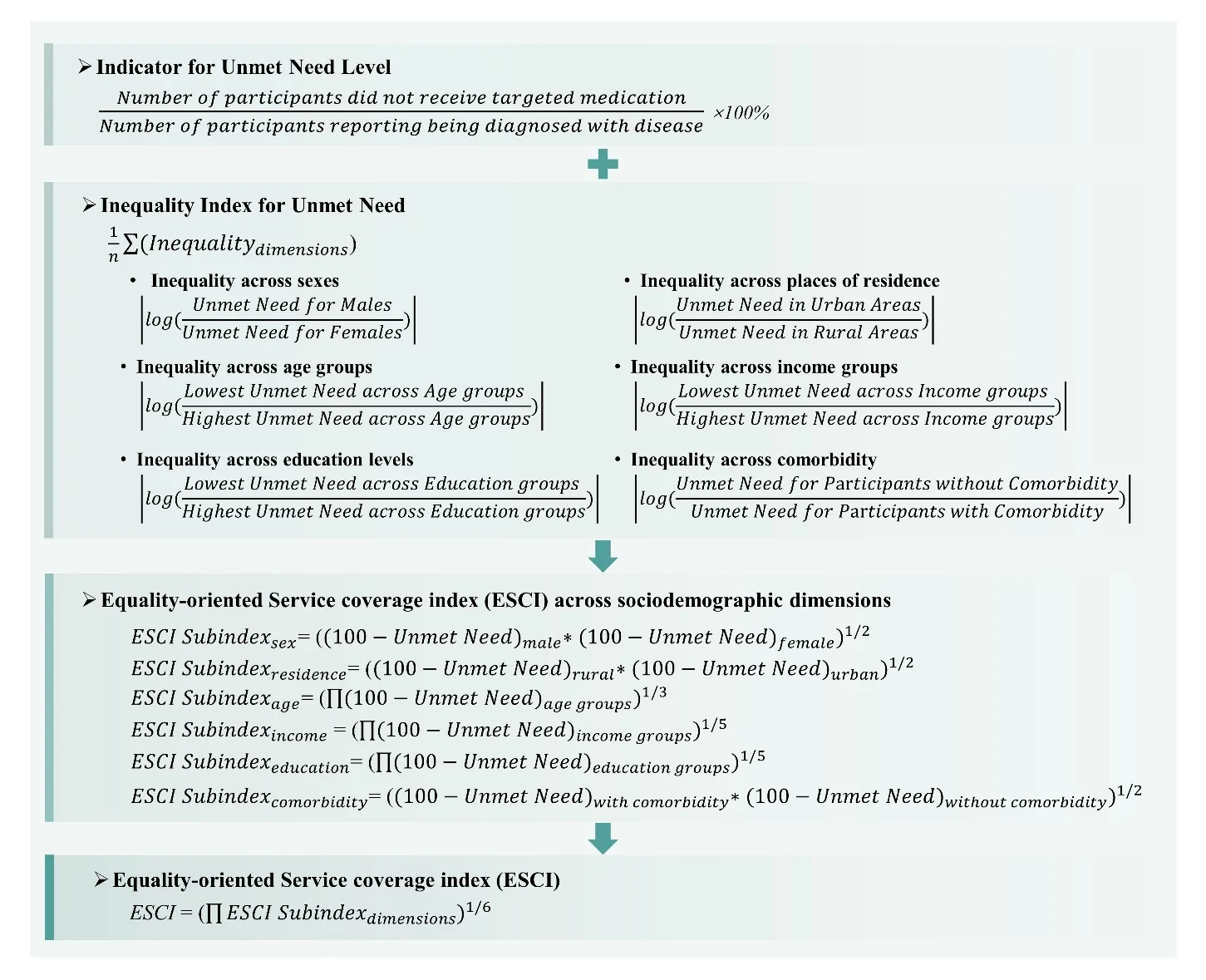
Conception and schematic framework of unmet needs level, inequality index, and equality-oriented service coverage index (ESCI).

Based on unmet needs level and inequality index, we established ESCI following the concept of the UHC service coverage index. A schematic of the concept of the ESCI sub-index and ESCI is shown in Figure 1. The ESCI is a non-scale index ranging from 0 to 100. A higher ESCI represents a lower level of unmet needs as well as lower inequality.

$${\mathrm{ESCI Sub\_index}}_{\mathrm{dimension}(i)}=({\prod {\left(100-\mathrm{Unmet Need}\right)}_{\mathrm{subgroups in dimensino}(i)})}^{1/n}$$

$${\mathrm{ESCI}=(\prod {\mathrm{ESCI Sub\_index}}_{\mathrm{dimensions}})}^{1/6}$$

*Mortality in older adults:* In this study, the primary health outcome was defined as all-cause mortality (per 100,000 population) in people over 50 years of age. The secondary health outcome was defined as the mortality rate owing to NCDs in older people. We further used the mortality rate due to the four chronic conditions as the health outcome variable. The data for mortality in older adults were estimated following the method applied in the Global Burden of Disease (GBD) study.

### Statistical analysis

The level and 95% confidence interval (CI) of unmet medication needs were estimated by country and also across sociodemographic strata: sex (female vs. male), place of residence (rural vs. urban), age group (<60, 60-69, 70-79, ≥80 years), income (household income quintiles), and education level.

The association between ESCI and mortality was explored through a country-level ecological study design. ESCI and ESCI sub-index were separately defined as independent variables, and all-cause mortality as well as mortality owing to noncommunicable diseases (NCDs) in individuals over 55 years of age were defined as dependent variables. Two linear regression models were applied, in which model 1 included log-transformed gross national income (GNI) per capita and log-transformed proportion of older people out of total population; model 2 further included the proportion of health expenditure out of total government expenditure. In sensitivity analysis, a parallel association analysis was conducted only in 28 high-income countries.

All statistical analyses in this study were conducted using Stata version 14.0 (StataCorp LLC, College Station, TX, USA) and R v4.1.3 software (http://www.r-project.org/). All tests were two-sided and *P* values < 0.05 were considered statistically significant.

## RESULTS

### Unmet medication needs

**Figure 2.**
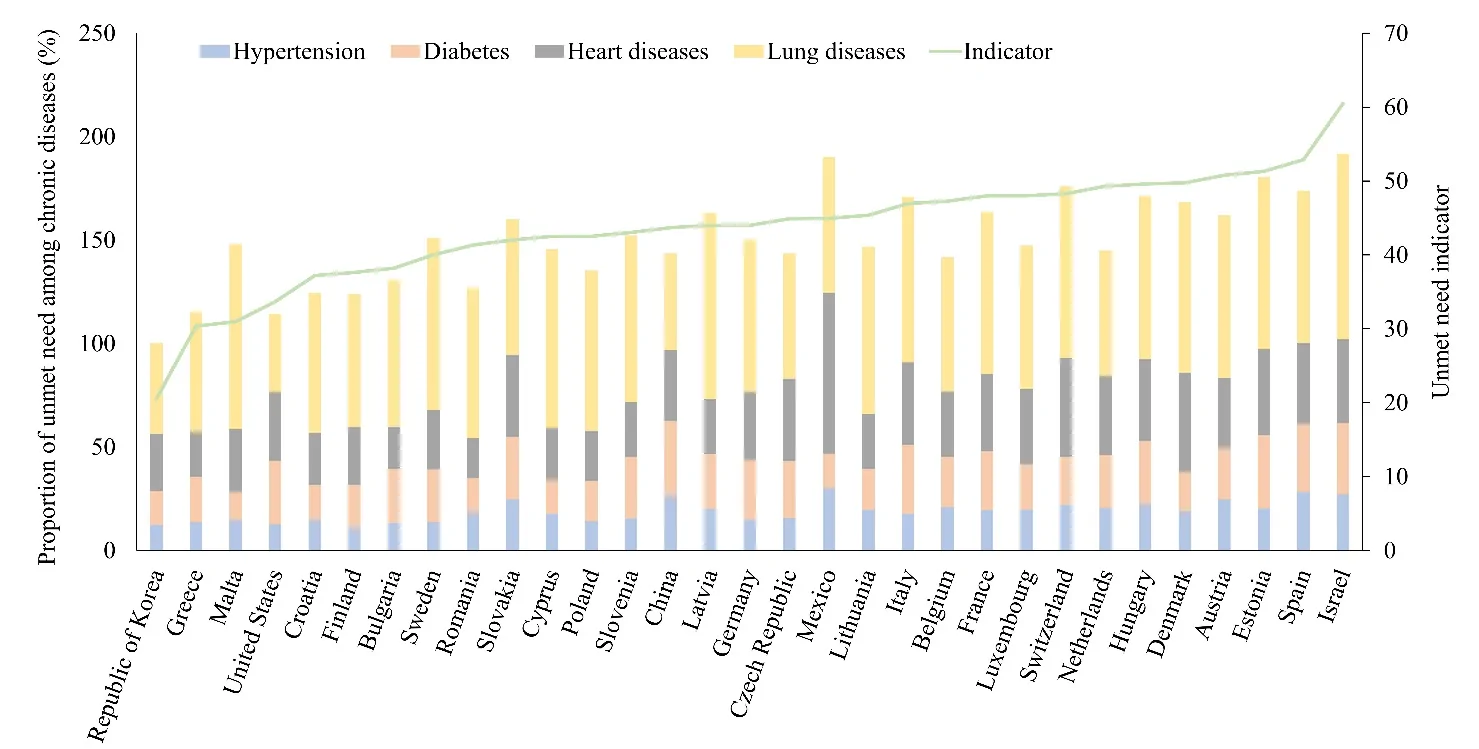
Unmet needs level and overall indicators according to chronic condition and country.

A total of 69,443 participants with at least one of four common chronic diseases were included in the analysis. 40,387 participants reported that they had received the targeted medication or treatments, leaving 29,056 participants with unmet medication needs. The overall unmet medication needs reached 41.84% (95% CI: 41.47%, 42.21%) for older people. The country-specific levels for unmet medication needs were depicted in Figure 2. Israel had the highest unmet needs level at 60.51%, and Republic of Korea had the lowest unmet needs level at 20.57%.

Unmet medication needs showed disparity across the four chronic diseases. Among 57,456 participants diagnosed with hypertension, 11,456 (19.94%, 95% CI: 19.61%, 20.27%) reported having an unmet medication need. This proportion was 25.80% (95% CI: 25.22%, 26.39%), 60.30% (95% CI: 59.43%, 61.17%), and 37.96% (95% CI: 37.31%, 38.62%) for diabetes, lung disease, and heart disease, respectively (Fig. 2).

### Unmet medication needs inequality and ESCI

**Figure 3.**
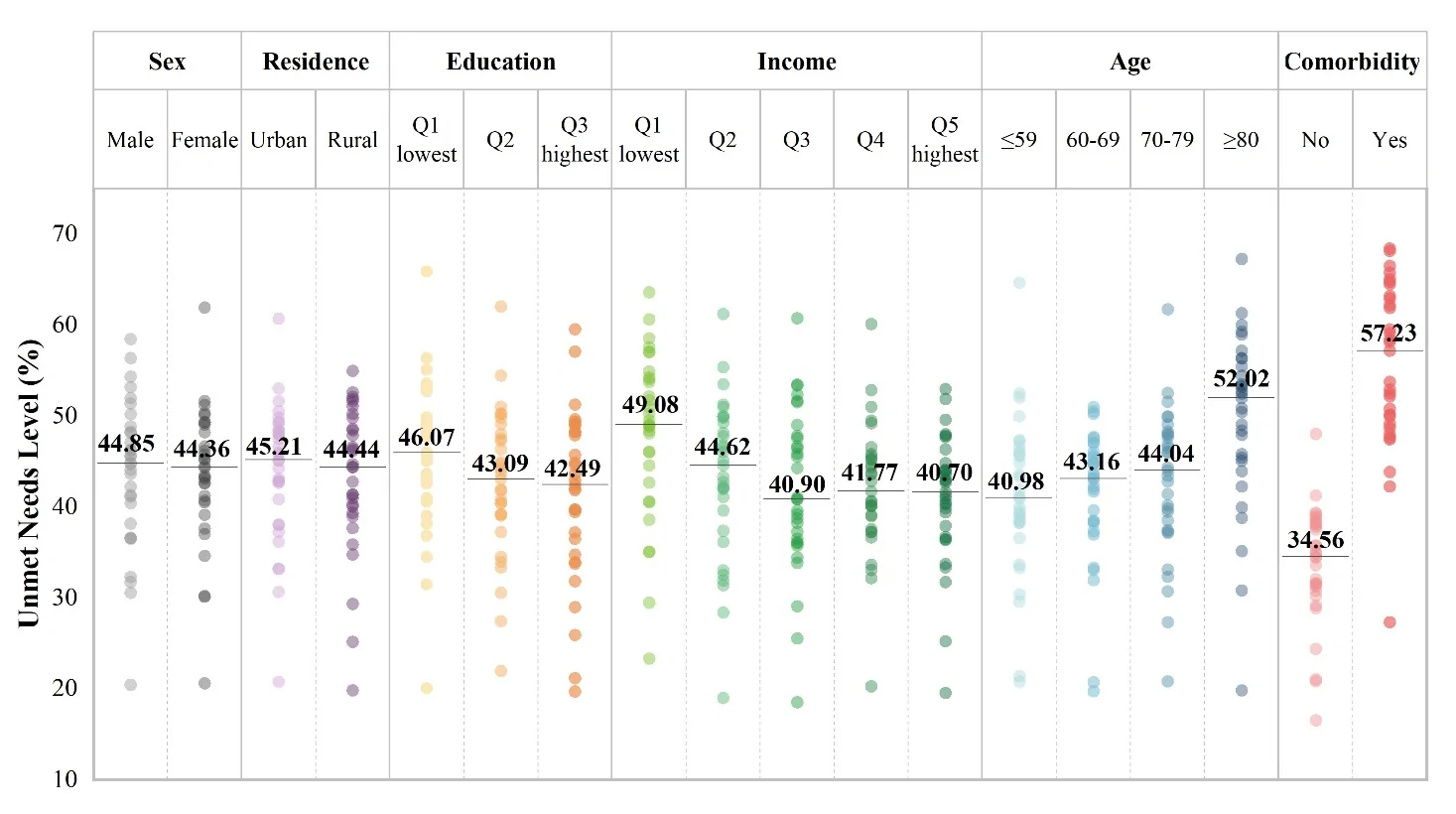
Unmet needs level across sociodemographic subgroups.

**Figure 4.**
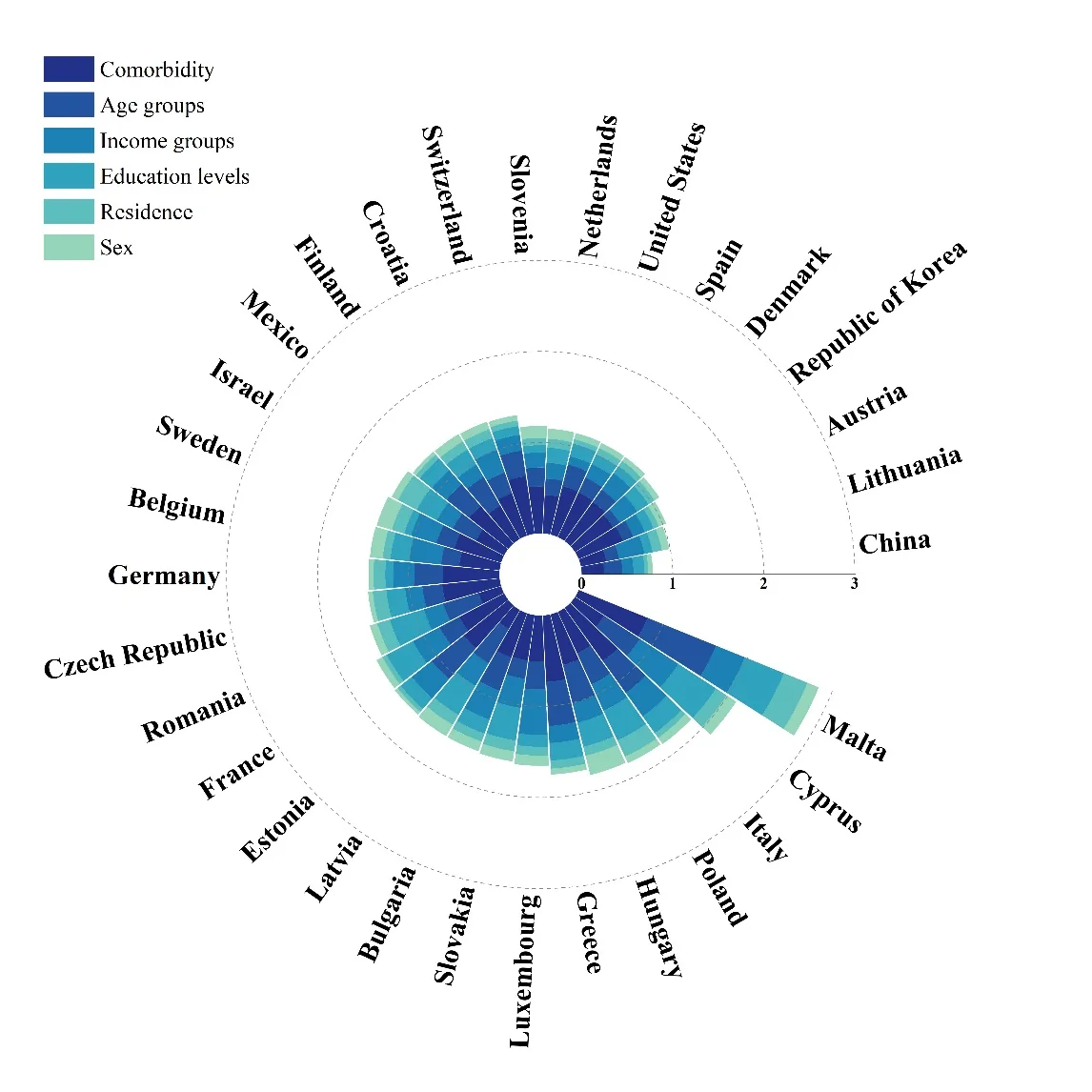
Relative inequality index of unmet needs for medication in older adults according to dimension and country.

Figure 3 showed the unmet medication needs level by sociodemographic strata. The median unmet needs level was 57.23% among participants with multimorbidity, which was over 1.5 times the level for participants without multimorbidity (34.56%). Unmet medication needs showed an increasing trend with age, with a level of 52.02% in participants over 80 years of age. Unmet medication needs decreased with higher educational level and income groups. Slightly higher unmet needs level for medication was observed for older men (44.85%) than older women (44.36%) and for participants with urban residence (45.21%) as compared with those living in rural areas (44.44%). An index reflecting the inequality across six sociodemographic dimensions was established (Fig. 4 and Supplementary Table 4). Multimorbidity and age group were the two leading contributors to the inequality index. Sensitivity analysis using disease-specific estimation showed robust results (Supplementary Fig. 1-3, Supplementary Table 5). Sensitivity analysis using individual level sampling weight reported similar results (Supplementary Table 6-7).

**Figure 5.**
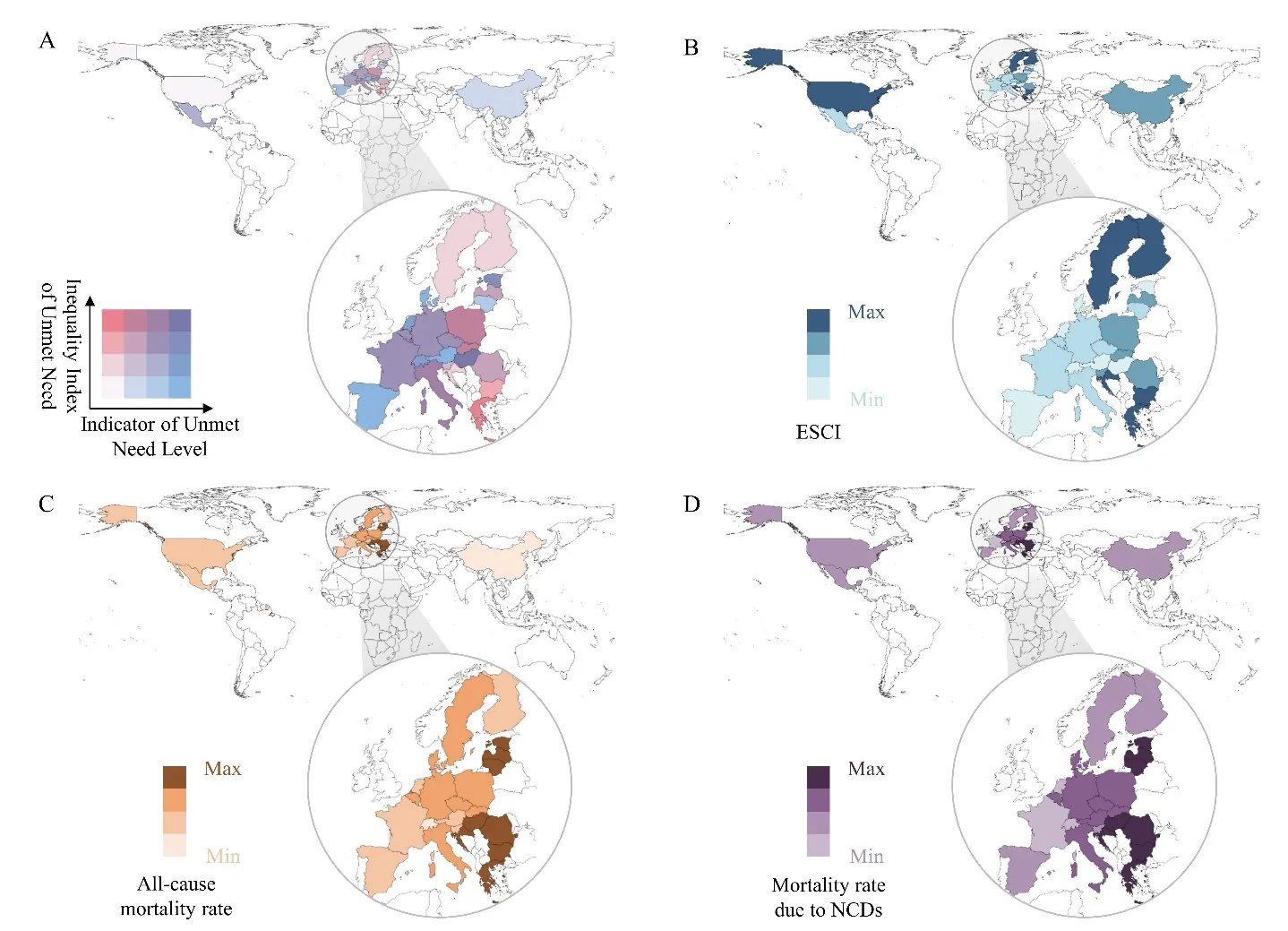
**Global map of unmet needs level and inequality for medication in older adults and map of equality-oriented service coverage index (ESCI) in 31 countries in the current study**. (**A**) Map of unmet needs level and inequality for medication; (**B**) Map of ESCI index; (**C**) Map of all-cause mortality rate; (**D**) Map of mortality rate due to noncommunicable diseases.

The performance of the ESCI in 31 countries across three continents was shown in Figure 5. The ESCI values reflected both the level of medication coverage and equality across sociodemographic subgroups (Supplementary Fig 4). Israel had the lowest ESCI value (40.07) and Republic of Korea had the highest (79.25) among the 31 countries included into current study. The ESCI sub-index across six sociodemographic strata was also calculated (Supplementary Table 8).

### Association of ESCI and mortality in older adults

**Figure 6.**
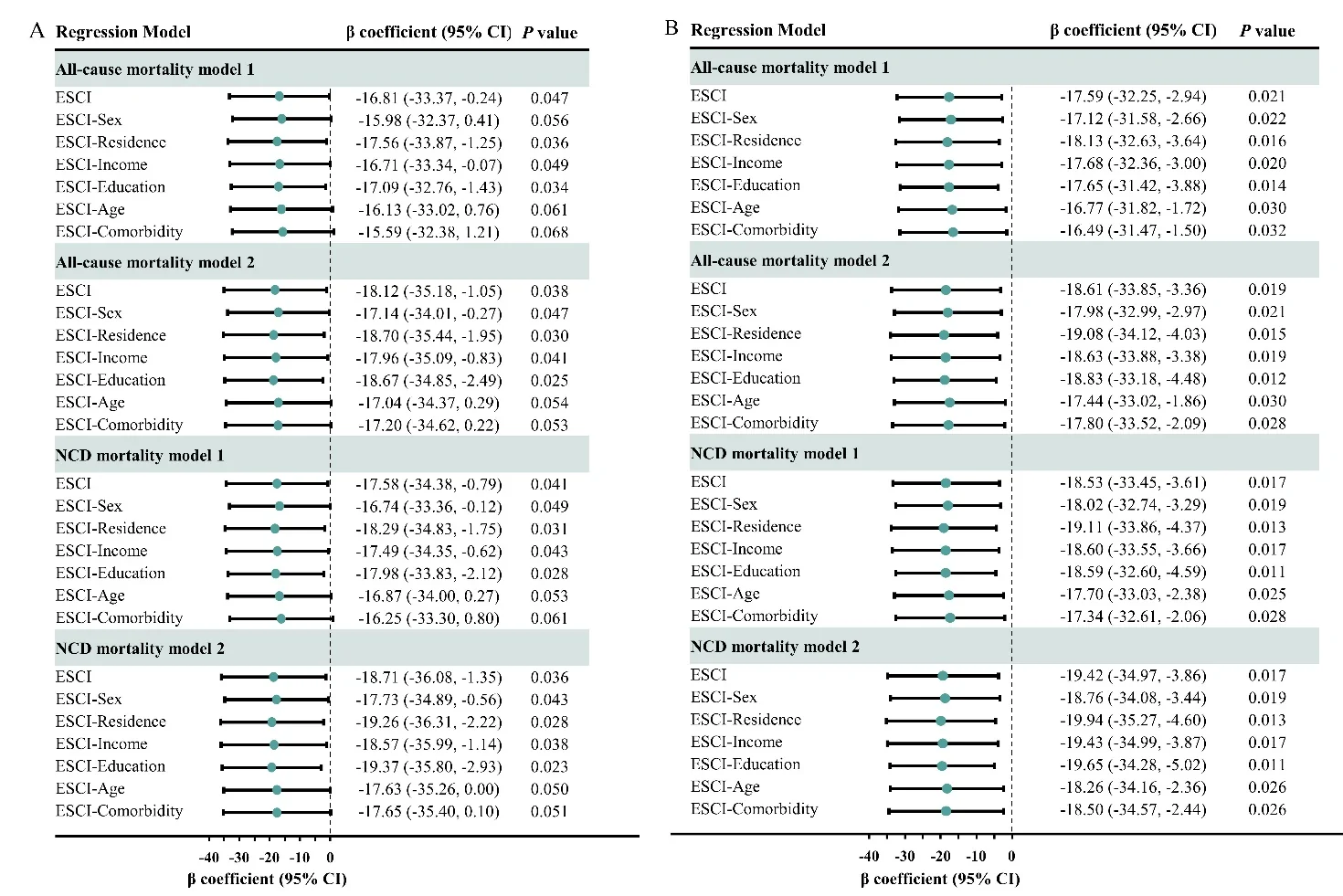
**Association of equality-oriented service coverage index (ESCI) and mortality rate in older adults**. (**A**) Association between ESCI and all-cause mortality rate; (**B**) Association between ESCI and mortality rate due to non-communicable diseases.

Figure 6 showed the β coefficient and 95% CI in the association analysis between the ESCI and sub-index with mortality in older adults. After adjusting for GNI per capital and proportion of the population aged 65 years and above out of the total population, the ESCI was a good predictor of all-cause mortality rate in older adults (β=-16.81, *P*=0.047, 95% CI: -33.37, -0.24) as well as mortality owing to NCDs (β=-17.58, *P*=0.041, 95% CI: -34.38, -0.79). The associations were robust after adjusting for the proportion of health expenditure out of total government expenditure (all-cause mortality: β=-18.12, *P*=0.038, 95% CI: -35.18, -1.05; NCD mortality: β=-18.71, *P*=0.036, 95% CI: -36.08, -1.35). The dimension-specific ESCI sub-index was also explored in association with mortality rate in older adults. ESCI sub-indexes according to sex, residence, income quintile, and education level were inversely associated with all-cause mortality and mortality rate owing to NCDs.

We further conducted analysis by applying the mortality rate due to the four chronic conditions as the outcome variable in association analysis (Supplementary Table 9). Analysis in 28 high-income countries also showed that ESCI was in statistically significant association with all-cause mortality (Figure 6B, β=-18.61, *P*=0.019, 95% CI: -33.85, -3.36) and mortality owing to NCDs (β=-19.42, *P*=0.017, 95% CI: -34.97, -3.86). ESCI sub-indexes across the six sociodemographic strata were inversely associated with health outcomes in older adults

## DISCUSSION

Countries worldwide are experiencing growth in both the number and proportion of people aged 60 years and older in the population, and all countries face major challenges to ensure UHC to respond to population aging. This study investigated the unmet medication needs in older individuals and developed an innovative indicator measuring unmet needs and inequality. Using multi-country population-based cohorts, we investigated the association between unmet needs and mortality in older adults.

Our results revealed that a concerning 41.84% of older adults with chronic conditions did not have their medication needs met, even in high-income countries. This observation emphasized the urgency to focus on barriers to medication accessibility to achieve health for all. European Union indicated that 27.1% of people aged 45-64 years and 23.9% of those aged 65 years or over have reported unmet needs for health care (https://ec.europa.eu/eurostat/statistics-explained/index.php?title=Unmet_health_care_needs_statistics). United States Health statistics in 2019 suggested that 12.2% individuals had delays in receiving or did not receive needed medical care in the previous 12 months (www.cdc.gov/nchs/hus/topics/unmet-need.htm). The unmet medication levels estimated in this study were higher than these health statistics owing to different definitions of unmet needs in the analyses. For example, the survey estimated the unmet healthcare need in Korea by asking “During the past 12 months, was there a time when you did not receive the medical service you needed?” [15]. In this study, we defined unmet needs in terms of medication and treatment of chronic conditions, rather than using self-report health status to evaluate unmet outpatient and inpatient care needs [16]. The unmet medication needs in the older population revealed the matters of healthcare system, especially the availability, accessibility, affordability, and acceptability of healthcare services for the older population [17]. Lung diseases had the highest level of unmet needs among the four chronic conditions. This phenomenon was similarly reported in a Brazil survey (8.5% for chronic respiratory disease vs. 2.1% for hypertension) [18]. It may be caused by suboptimal adherence to treatment owing to the need for an inhaled route of drug administration in lung disease management, indicating the need for patient-tailored inhalers, electronic monitoring, and the use of digital technology to improve medication accessibility.

The unmet needs varied across sociodemographic strata. Participants with older age and multimorbidity had a much greater likelihood of having unmet medication needs. Patients with multimorbidity would have a higher risk of physical disability [19], which could be a barrier to the utilization of health care services [20]. The inequality index was inversely associated with the UHC index of service capacity and access, revealing that more equitable health care service utilization could be achieved through strengthened health care service delivery systems. We proposed that equality-oriented monitoring of progress toward UHC could comprise complementary dimensions of inequality, and a whole-spectrum approach examining both the level and inequality should be adopted.

By including five cohorts from 31 countries, we identified an inverse association between ESCI and all-cause mortality and mortality owing to NCDs in participants over 55 years of age. This finding indicated that ESCI could serve as an evaluation indicator to monitor the progress toward UHC and the healthy aging agenda. Among all health care services, medications were the most important therapeutic tools, as well as a core determinant of health outcomes and healthy aging [21-23]. Enormous gaps remained in improving access to appropriate medication services, which were listed as priorities in pre- and post-marketing phases of drug research [24]. In this study, we first established an association between equality-oriented health care service coverage and health consequences. It provided evidence to support the concept that every subgroup should be valued, and that no one should be left behind in the process of achieving UHC and the 2030 SDG health targets.

UHC cannot be achieved without considering the unmet needs of older individuals and rapidly accelerating population aging [25]. In 2016, the World Health Assembly adopted the "Global Strategy and Action Plan on Aging and Health for 2016-2020," and emphasized the alignment of health systems with the needs of older people, as well as improving the measurement, monitoring, and research on healthy aging (www.who.int/publications/i/item/9789241513500). In the first United Nations (UN) Decade of Healthy Aging progress report released by WHO and UN partners, delivering person-centered, integrated care and primary health services that are responsive to the needs of older people is a key area of action (www.who.int/publications/i/item/9789240079694). Under this context, results from the current study delivered evidence-based policy and practical implications for tackling the progress toward UHC as well as healthy aging. For example, conducting regular surveys and measuring unmet aging-suited health care needs and the ESCI could be implemented. This approach can be used to identify vulnerable populations with greater unmet needs, and to reveal directions to establish an aging-appropriate health system to meet the needs of older adults.

This study has several strengths. First, we measured unmet medication needs among older adults by using a clear definition which was comparable across countries and regions. Second, we measured unmet medication needs in 31 countries across three continents using national surveys in studies recruiting large representative samples. Third, we identified the association between ESCI and health outcomes and discussed evidence-based policy implications for process evaluation in monitoring progress toward UHC and healthy aging. This study also had certain limitations. First, this study used an ecological study design to conduct association analysis by linking the unmet needs with mortality rate at country-level. The association at individual level could be further explored. Second, even though 31 countries were included in the current analysis, low- and middle-income countries were underrepresented. Third, we could not capture the reasons for unmet needs or forgone care owing to a lack of data availability. Further surveys focused on the barriers and facilitators of unmet medication needs, especially in low- and middle-income countries, would provide valuable data to measure unmet medication needs and equality-oriented health care coverage.

In conclusion, an alarming 41.84% of older adults with chronic conditions had unmet needs for medication, and the unmet needs were highest in participants >80 years of age and those with multimorbidity. The equality-oriented service coverage index was inversely associated with mortality in older adults. Our findings reinforce the importance of measuring unmet needs and equality-oriented health care service coverage in older adults, which can enrich the framework of UHC, and of monitoring the progress toward healthy aging and SDG health targets.

## Supplementary Materials

The Supplementary data can be found online at: www.aginganddisease.org/EN/10.14336/AD.2024.0626.

## Data Sharing

The data that support the findings of this study are available from the GATEWAY TO GLOBAL AGING DATA (https://g2aging.org/).
