# Supplementary material for: Unmet Medication Needs and the Association with Mortality in Older Adults: Equality-Oriented Monitoring toward Universal Health Coverage
Source: Aging Dis. 2024 Aug 6;16(4):2398–407. doi: 10.14336/AD.2024.0626 (PMC12221402; doi:10.14336/AD.2024.0626)
Supplement: Supplementary file 1 — The Supplementary data can be found online at: www.aginganddisease.org/EN/10.14336/AD.2024.0626. [file AD-16-4-2398-s.pdf]

SUPPLEMENTARY DATA

Unmet Medication Needs and the Association with Mortality in Older Adults: Equality-Oriented Monitoring toward Universal Health Coverage

Minmin Wang, Yikai Feng, Yanan Luo, Yinzi Jin, Minghui Ren, Zhi-Jie Zheng

© 2024. Wang M. et al. Published online at http://www.aginganddisease.org/EN/10.14336/AD.2024.0626

SUPPLEMENTARY DATA

Supplementary Table 1. Information of variables used in indicator validation and association analysis.

Variable	Classification of the variable	Coding forms in this study	Data source
UHC Service Coverage sub-index on service capacity and access	Country-level	Continuous variable	WHO Global Health Observatory (https://www.who.int/data/gho)
Age standardized all-cause mortality in population over 55 years of age	Country-level	Continuous variable	GBD 2019 (https://vizhub.healthdata.org/gbd-results/)
Age standardized mortality due to noncommunicable diseases in population over 55 years of age	Country-level	Continuous variable	GBD 2019 (https://vizhub.healthdata.org/gbd-results/)
GNI per capital	Country-level	Log-transformed continuous variable	World Bank Open Data (https://data.worldbank.org.cn/)
Proportion of population aged 65 years and above out of the total population	Country-level	Log-transformed continuous variable	World Bank Open Data (https://data.worldbank.org.cn/)
Proportion of health expenditure out of the total government expenditure	Country-level	Continuous variable	World Bank Open Data (https://data.worldbank.org.cn/)

Abbreviations: UHC, universal health coverage; WHO, World Health Organization; GBD, Global Burden of Disease; GNI, gross national income

Supplementary Table 2. Details of questions used to determine participants' needs in the HRS, SHARE, CHARLS, MHAS, and KLoSA surveys

Database	Variable	Label	Questions
HRS*	qc005	HIGH BLOOD PRESSURE	Has a doctor ever told you that you have high blood pressure or hypertension? /Since we last asked you (in R's LAST IW MONTH, YEAR), has a doctor told you that you have high blood pressure or hypertension?
	qc010	DIABETES	Has a doctor ever told you that you have diabetes or high blood sugar? /Since we last asked you in R's LAST IW MONTH, YEAR, has a doctor told you that you have diabetes or high blood sugar?
	qc030	LUNG DISEAS	Has a doctor ever told you that you have chronic lung disease such as chronic bronchitis or emphysema? / (Since we last asked you (in R's LAST IW MONTH, YEAR),) has a doctor told you that you have chronic lung disease, such as chronic bronchitis or emphysema?
	qc036	HEART CONDITION	Has a doctor ever told you that you have had a heart attack, coronary heart disease, angina, congestive heart failure, or other heart problems? / (Since we last asked you (in R's LAST IW MONTH, YEAR) has a doctor told you that you have had) A heart attack, (have) coronary heart disease, angina, congestive heart failure, or other heart problems?

© 2024. Wang M. et al. Published online at http://www.aginganddisease.org/EN/10.14336/AD.2024.0626

SHARE	R8HIBPE	w8 R ever had high blood pressure	Whether a doctor has told the respondent they had or currently have high blood pressure or hypertension?
	R8DIABE	w8 R ever had diabetes	Whether a doctor has told the respondent they had or currently have diabetes or high blood sugar?
	R8LUNGE	w8 R ever had lung disease	Whether a doctor has told the respondent they had or currently have chronic lung disease such as chronic bronchitis or emphysema?
	R8HEARTE	w8 R ever had heart problems	Whether a doctor has told the respondent they had or currently have a heart attack, including myocardial infarction or coronary thrombosis, or any other heart problem, including congestive heart failure?
CHARLS	R4HIBPE	w4 r Ever had high blood pressure	Whether the respondent reported having hypertension?
	R4DIABE	w4 r ever had diabetes	Whether the respondent reported having diabetes or high blood sugar?
	R4LUNGE	w4 r ever had lung disease	Whether the respondent reported having chronic lung disease such as chronic bronchitis or emphysema (excluding tumors, or cancer)?
	R4HEARTE	w4 r ever had heart problem	Whether the respondent reported having heart attack, coronary heart disease, angina, congestive heart failure, or other heart problems?
MHAS	R5HIBPE	w5 R Ever had high blood pressure	Whether a doctor has told the respondent they had or currently have hypertension or high blood pressure?
	R5DIABE	w5 R Ever had diabetes	Whether a doctor has told the respondent they had or currently have diabetes or high blood sugar?
	R5RESPE	w5 R Ever had respiratory disease, incl asthma	Whether a doctor has told the respondent they had or currently have a respiratory illness, such as asthma or emphysema?
	R5HEARTE	w5 R Ever had heart problems	Whether a doctor has told the respondent they had a heart condition, such as heart failure/cardiac failure/congestive heart failure, arrhythmia, angina, or a heart attack?
KLoSA	R7HIBPE	w7 R ever had high BP	Whether the respondent has ever been diagnosed by a doctor as having high blood pressure?
	R7DIABE	w7 R ever had diabetes	Whether the respondent has ever been diagnosed by a doctor as having diabetes or high blood sugar?
	R7LUNGE	w7 R ever had lung disease	Whether the respondent has ever been diagnosed by a doctor as having lung disease (such as bronchitis or emphysema)?
	R7HEARTE	w7 R ever had heart problem	Whether the respondent has ever been diagnosed by a doctor as having heart problems (such as having had a heart attack, coronary heart disease, angina, congestive heart failure)?

*Combined with variables from Harmonized HRS and RAND HRS Fat File.

Abbreviations: HRS, Health and Retirement Study; SHARE, Survey of Health, Ageing and Retirement in Europe; CHARLS, China Health and Retirement Longitudinal Study; MHAS, Mexican Health and Aging Study; KLoSA, Korean Longitudinal Study of Aging.

© 2024. Wang M. et al. Published online at http://www.aginganddisease.org/EN/10.14336/AD.2024.0626

SUPPLEMENTARY DATA

Supplementary Table 3. Details of questions used to determine whether participants' needs were met in the HRS, SHARE, CHARLS, MHAS, and KLoSA surveys

Database	Variable	Label	Questions
HRS*	R14RXHIBP	w14 r takes meds for high blood pressure	Whether the respondent takes medication for high blood pressure?
	R14RXDIAB	w14 r takes meds for diabetes	Whether the respondent either takes oral medication or uses insulin shots for diabetes?
	R14RXLUNG	w14 r takes meds for lung condition	Whether the respondent takes medication for chronic lung disease, such as chronic bronchitis or emphysema, not including asthma?
	R14RXHEART	w14 r takes meds for heart problems	Whether the respondent takes medication for a heart attack or myocardial infarction?
SHARE	R8RXHIBP	w8 R takes meds for high BP	Whether the respondent takes medication for high blood pressure?
	R8RXDIAB	w8 R takes meds for diabetes	Whether the respondent takes medication for diabetes?
	R8RXLUNG	w8 R takes meds for lung condition	Whether the respondent takes medication for chronic bronchitis?
	R8RXHEART	w8 R takes meds for heart problem	Whether the respondent takes medication for heart problems?
CHARLS	R4RXHIBP_C	w4 r takes any meds for high blood pressure	Whether the respondent takes any medication for high blood pressure, which includes Western modern medicine or Chinese traditional medicine?
	R4RXDIAB_C	w4 r takes any meds for diabetes	Whether the respondent is taking any medication for diabetes, which includes insulin injections, Western modern medicine, or Chinese traditional medicine?
	R4RXLUNG_C	w4 r takes any meds for lung condition	Whether the respondent takes any medication, which includes Western modern medicine or Chinese traditional medicine, for chronic lung diseases, such as chronic bronchitis, emphysema (excluding tumors, or cancer)?
	R4RXHEART_C	w4 r takes any meds for heart problems	Whether the respondent takes any medication, which includes, Western modern medicine and Chinese traditional medicine for heart attack, coronary heart disease, angina, congestive heart failure, or other heart problems?
MHAS	R5RXHIBP	w5 Whether R takes meds for high blood pressure	Whether the respondent takes medication for hypertension or high blood pressure?
	R5RXDIAB	w5 Whether R takes meds for diabetes (oral or insulin)	Whether the respondent uses any medication (oral medication or insulin shots) for diabetes?
	R5RXRESP	w5 Whether S takes meds for respiratory disease	Whether the respondent takes medication for a respiratory illness, such as asthma or emphysema?

© 2024. Wang M. et al. Published online at http://www.aginganddisease.org/EN/10.14336/AD.2024.0626

	R5RXHRTAT	w5 Whether R takes meds for heart attack	Whether the respondent takes medication for a heart attack?
KLoSA	R7RXHIBP	w7 R takes meds/treatment for high BP	Whether the respondent takes medication or is receiving treatment for high blood pressure?
	R7RXDIAB	w7 R takes meds/treatment for diabetes	Whether the respondent takes medication or is receiving treatment for diabetes?
	R7RXLUNG	w7 R takes meds/treatment for lung disease	Whether the respondent takes medication or is receiving treatment for lung disease?
	R7RXHEART	w7 R takes meds/treatment for heart problem	Whether the respondent takes medication or is receiving treatment for heart problems, including heart attack, angina pectoris, myocardial infarction, congestive heart failure, or other heart disease?

*Combined with variables from Harmonized HRS and RAND HRS Fat File.

Abbreviations: HRS, Health and Retirement Study; SHARE, Survey of Health, Ageing and Retirement in Europe; CHARLS, China Health and Retirement Longitudinal Study; MHAS, Mexican Health and Aging Study; KLoSA, Korean Longitudinal Study of Aging.

© 2024. Wang M. et al. Published online at http://www.aginganddisease.org/EN/10.14336/AD.2024.0626

SUPPLEMENTARY DATA

Supplementary Table 4. Relative inequality index of unmet needs for medication in older adults according to dimension and country.

Country	Age groups	Comor bidity	Education levels	Income groups	Place of residence	Sex	Relative inequality index
Austria	0.12	0.52	0.10	0.14	0.06	0.09	0.17
Belgium	0.20	0.45	0.20	0.36	0.09	0.14	0.24
Bulgaria	0.39	0.52	0.16	0.33	0.09	0.11	0.27
China	0.20	0.25	0.12	0.12	0.04	0.05	0.13
Croatia	0.24	0.49	0.13	0.27	0.10	0.09	0.22
Cyprus	0.47	0.38	0.49	0.50	0.14	0.12	0.35
Czech Republic	0.22	0.64	0.21	0.19	0.15	0.05	0.24
Denmark	0.17	0.53	0.09	0.22	0.07	0.05	0.19
Estonia	0.45	0.63	0.15	0.28	0.04	0.04	0.27
Finland	0.36	0.43	0.19	0.16	0.10	0.09	0.22
France	0.25	0.55	0.33	0.33	0.05	0.07	0.26
Germany	0.31	0.62	0.09	0.23	0.13	0.06	0.24
Greece	0.48	0.73	0.19	0.19	0.09	0.06	0.29
Hungary	0.44	0.57	0.27	0.22	0.10	0.22	0.30
Israel	0.29	0.35	0.25	0.20	0.23	0.08	0.23
Italy	0.43	0.72	0.23	0.32	0.06	0.08	0.31
Latvia	0.44	0.24	0.37	0.29	0.13	0.14	0.27
Lithuania	0.14	0.29	0.09	0.29	0.10	0.08	0.17
Luxembourg	0.31	0.51	0.13	0.50	0.10	0.11	0.28
Malta	0.81	0.82	0.46	0.34	0.28	0.13	0.47
Mexico	0.32	0.55	0.17	0.21	0.05	0.04	0.22
Netherlands	0.18	0.42	0.17	0.17	0.10	0.11	0.19
Poland	0.38	0.50	0.36	0.32	0.20	0.08	0.31
Republic of Korea	0.09	0.50	0.15	0.23	0.08	0.06	0.18
Romania	0.41	0.26	0.39	0.22	0.14	0.09	0.25
Slovakia	0.20	0.51	0.36	0.32	0.12	0.11	0.27
Slovenia	0.21	0.52	0.08	0.16	0.08	0.13	0.20
Spain	0.18	0.54	0.12	0.16	0.07	0.06	0.19
Sweden	0.20	0.54	0.08	0.29	0.14	0.17	0.24
Switzerland	0.29	0.64	0.10	0.17	0.06	0.06	0.22
United States	0.24	0.55	0.14	0.09	0.05	0.07	0.19

© 2024. Wang M. et al. Published online at http://www.aginganddisease.org/EN/10.14336/AD.2024.0626

Supplementary Table 5. Absolute inequality index of unmet needs for medication in older adults according to dimension and country.

Country	Age groups	Comor bidity	Education levels	Income groups	Place of residence	Sex	Absolute inequality index
Austria	6.35	26.40	5.27	6.94	3.06	4.49	8.75
Belgium	9.66	21.41	9.65	16.93	3.94	6.60	11.37
Bulgaria	13.83	19.78	6.38	12.50	3.64	4.14	10.04
China	8.52	11.03	5.37	5.26	1.75	2.15	5.68
Croatia	9.27	18.48	4.75	10.04	3.77	3.27	8.26
Cyprus	16.49	16.01	22.00	20.45	6.18	5.22	14.39
Czech Republic	10.19	26.88	9.39	8.49	6.99	2.28	10.70
Denmark	8.49	27.08	4.87	11.35	3.33	2.73	9.64
Estonia	21.50	30.02	8.04	14.02	2.21	2.03	12.97
Finland	14.29	16.65	7.08	6.11	3.91	3.48	8.59
France	12.31	26.45	15.25	15.31	2.42	3.52	12.54
Germany	13.98	27.39	3.77	10.17	5.52	2.47	10.55
Greece	13.27	22.77	5.46	5.82	2.64	1.94	8.65
Hungary	19.39	26.83	14.25	10.74	5.10	11.00	14.55
Israel	16.55	20.16	14.37	11.10	12.42	4.60	13.20
Italy	20.66	33.37	9.87	14.65	2.86	3.96	14.23
Latvia	18.38	10.75	16.19	12.82	5.38	6.06	11.60
Lithuania	6.36	13.02	4.05	13.17	4.57	3.80	7.50
Luxembourg	16.21	24.92	6.32	23.45	4.57	5.35	13.47
Malta	25.05	26.59	11.55	10.09	7.90	4.14	14.22
Mexico	15.41	24.56	7.92	9.57	2.73	1.97	10.36
Netherlands	8.36	21.81	8.33	8.60	5.01	5.44	9.59
Poland	16.58	20.62	14.55	13.62	8.26	3.37	12.83
Republic of Korea	1.77	10.78	3.01	4.74	1.60	1.27	3.86
Romania	16.72	11.17	12.61	9.07	6.26	3.55	9.90
Slovakia	8.58	23.11	13.37	13.41	5.04	4.71	11.37
Slovenia	9.24	21.65	3.54	7.22	3.38	5.77	8.47
Spain	9.45	27.68	5.93	8.81	3.73	3.25	9.81
Sweden	8.43	22.06	3.15	12.15	5.24	6.95	9.66
Switzerland	12.48	32.19	4.72	8.30	2.95	2.89	10.59
United States	8.47	17.86	4.92	3.17	1.67	2.29	6.40

© 2024. Wang M. et al. Published online at http://www.aginganddisease.org/EN/10.14336/AD.2024.0626

Supplementary Table 6. The unmet medication needs in 31 countries by using the individual level weight in five cohorts.

	All	Hypertension	Diabetes	Heart diseases	Lung diseases
America	32.67	11.35	28.57	32.40	37.64
Austria	55.75	25.88	28.88	40.11	89.12
Belgium	47.85	21.07	27.96	30.47	67.31
Bulgaria	36.55	14.21	25.29	18.01	72.07
China	44.30	26.64	35.76	34.49	49.73
Croatia	39.18	16.06	16.25	25.98	66.74
Cyprus	39.41	18.62	12.19	22.93	86.43
Czech Republic	41.06	15.36	25.62	42.43	57.90
Denmark	50.31	20.41	20.02	47.91	80.34
Estonia	48.75	20.96	35.14	42.23	84.26
Finland	40.79	14.22	19.80	33.52	78.23
France	48.73	20.59	30.59	34.28	79.98
Germany	43.90	15.09	31.18	31.28	76.03
Greece	29.28	13.51	20.62	21.66	57.17
Hungary	46.24	17.99	30.24	38.55	80.55
Israel	56.36	26.84	28.82	52.40	89.63
Italy	44.55	17.96	32.57	43.42	79.43
Latvia	43.13	20.15	28.78	29.42	90.32
Lithuania	44.42	20.58	18.17	27.17	82.53
Luxembourg	51.40	23.70	20.37	38.62	72.81
Malta	30.57	15.53	13.81	31.56	85.44
Mexico	48.55	34.54	18.71	78.23	70.17
Netherlands	44.48	21.08	23.26	31.53	57.03
Poland	42.49	14.92	19.83	26.77	77.88
Romania	40.38	20.01	16.20	17.15	75.19
Republic of Korea	20.38	0.00	17.46	28.00	45.84
Slovakia	42.07	22.75	29.03	41.59	70.14
Slovenia	44.16	18.00	29.12	25.81	82.53
Spain	53.07	26.44	35.27	34.63	79.74
Sweden	37.97	14.73	28.51	30.15	77.91
Switzerland	48.84	25.19	24.96	50.26	87.70

© 2024. Wang M. et al. Published online at http://www.aginganddisease.org/EN/10.14336/AD.2024.0626

Supplementary Table 7. The inequality index of unmet medication needs in 31 countries by using the individual level weight in five cohorts.

Country	Age groups	Education levels	Income groups	Comorbidity	Place of residence	Sex	Relative inequality index
America	0.14	0.08	0.19	0.74	0.08	0.05	0.21
Austria	0.19	0.04	0.47	0.49	0.09	0.07	0.23
Belgium	0.19	0.08	0.40	0.44	0.06	0.25	0.24
Bulgaria	0.34	0.14	0.35	0.48	0.07	0.15	0.26
China	0.22	0.26	0.10	0.23	0.03	0.06	0.15
Croatia	0.22	0.17	0.26	0.40	0.04	0.04	0.19
Cyprus	0.43	0.41	0.65	0.46	0.12	0.03	0.35
Czech Republic	0.27	0.24	0.35	0.54	0.18	0.17	0.29
Denmark	0.14	0.10	0.21	0.56	0.04	0.04	0.18
Estonia	0.44	0.21	0.38	0.64	0.04	0.01	0.29
Finland	0.43	0.38	0.31	0.41	0.07	0.34	0.32
France	0.27	0.39	0.54	0.47	0.01	0.01	0.28
Germany	0.36	0.12	0.25	0.64	0.14	0.03	0.26
Greece	0.42	0.26	0.13	0.73	0.05	0.02	0.27
Hungary	0.69	0.32	0.50	0.68	0.13	0.21	0.42
Israel	0.46	0.20	0.77	0.53	0.20	0.15	0.38
Italy	0.47	0.53	0.45	0.76	0.04	0.03	0.38
Latvia	0.44	0.39	0.30	0.32	0.07	0.10	0.27
Lithuania	0.15	0.06	0.17	0.29	0.08	0.11	0.14
Luxembourg	0.37	0.14	0.62	0.45	0.03	0.11	0.29
Malta	0.95	0.77	0.48	0.86	0.42	0.01	0.58
Mexico	0.21	0.35	0.16	0.29	0.07	0.05	0.19
Netherlands	0.71	0.29	0.23	0.30	0.02	0.00	0.26
Poland	0.36	0.37	0.31	0.62	0.10	0.01	0.30
Romania	0.49	0.53	0.28	0.28	0.12	0.08	0.30
Republic of Korea	0.05	0.14	0.32	0.43	0.03	0.04	0.17
Slovakia	0.28	0.48	0.29	0.56	0.02	0.06	0.28
Slovenia	0.24	0.10	0.20	0.41	0.02	0.04	0.17
Spain	0.26	0.64	0.28	0.54	0.00	0.03	0.29
Sweden	0.47	0.14	0.25	0.63	0.27	0.41	0.36
Switzerland	0.10	0.28	0.28	0.67	0.05	0.06	0.24

© 2024. Wang M. et al. Published online at http://www.aginganddisease.org/EN/10.14336/AD.2024.0626

SUPPLEMENTARY DATA

Supplementary Table 8. Dimension-specific equality-oriented service coverage index (ESCI) according to country.

**Country**	ESCI	ESCI-Sex	ESCI-Residence	ESCI-Income	ESCI-Education	ESCI-Age	ESCI-Comorbidity
Austria	48.52	48.80	48.89	49.32	49.07	48.47	46.60
Belgium	52.57	52.87	53.86	53.05	53.16	52.54	50.05
Bulgaria	61.52	62.08	61.48	62.05	61.39	62.17	59.97
China	56.27	56.23	56.45	56.73	55.52	57.50	55.22
Croatia	62.40	62.86	63.04	62.52	63.33	61.56	61.10
Cyprus	56.90	57.67	56.32	57.41	54.26	59.12	56.73
Czech Republic	54.54	54.99	53.55	55.12	54.49	54.48	54.61
Denmark	49.39	50.21	50.73	50.61	49.63	49.48	45.86
Estonia	48.94	48.67	48.60	49.51	48.25	50.42	48.20
Finland	61.45	62.36	61.65	62.49	62.09	60.38	59.76
France	51.81	52.16	52.02	52.44	53.07	51.96	49.26
Germany	55.55	55.99	56.86	56.00	55.99	55.75	52.81
Greece	69.47	69.62	69.96	69.36	70.60	70.82	66.54
Hungary	49.51	48.82	50.00	50.86	46.91	50.81	49.78
Israel	40.07	39.72	42.03	40.08	39.78	38.43	40.46
Italy	52.94	52.89	54.08	53.23	55.43	53.41	48.86
Latvia	55.99	56.83	56.88	55.95	55.29	56.05	54.98
Lithuania	54.47	54.16	55.21	54.66	54.66	54.55	53.63
Luxembourg	50.93	52.05	52.25	52.24	51.69	49.53	47.96
Malta	68.33	68.98	70.66	69.34	72.06	64.92	64.36
Mexico	52.06	52.77	49.19	53.12	53.41	51.33	52.68
Netherlands	49.66	50.57	49.41	50.76	49.80	51.11	46.45
Poland	57.42	57.29	57.59	57.59	58.57	56.33	57.21
Republic of Korea	79.25	79.44	79.66	79.81	79.38	79.41	77.83
Romania	58.44	58.49	56.87	58.65	62.23	58.04	56.56
Slovakia	57.00	58.09	57.71	58.62	59.24	56.71	51.94
Slovenia	56.74	56.51	57.12	57.12	56.95	56.77	56.01
Spain	47.35	46.95	48.29	47.35	48.97	47.36	45.28
Sweden	59.73	59.89	61.55	60.24	60.02	59.72	57.04
Switzerland	50.67	51.73	51.52	51.72	51.62	52.65	45.18
United States	66.22	66.49	65.97	66.51	65.93	66.41	66.04

© 2024. Wang M. et al. Published online at http://www.aginganddisease.org/EN/10.14336/AD.2024.0626

SUPPLEMENTARY DATA

Supplementary Table 9. Association between ESCI index and mortality rate due to the four types of chronic conditions in population over 55 years old.

	Model 1*	Model 2**
ESCI	-11.78 (-31.59, 8.03)	-12.44 (-33.04, 8.15)
ESCI-hypertension	-5.72 (-41.80, 30.36)	-6.44 (-44.32, 31.44)
ESCI-diabetes	-8.69 (-31.81, 14.43)	-8.68 (-32.43, 15.07)
ESCI-heart diseases	-0.74 (-17.41, 15.94)	-0.71 (-17.75, 16.33)
ESCI-lung diseases	-4.14 (-15.30, 7.03)	-4.68 (-16.59, 7.22)

ESCI, equality-oriented service coverage index.
* Model 1 included the log-transformed gross national income (GNI) per capita and log-transformed proportion of older people out of the total population.
** Model 2 further included the proportion of health expenditure out of the total government expenditure.

© 2024. Wang M et al. Published online at http://www.aginganddisease.org/EN/10.14336/AD.2024.0626

SUPPLEMENTARY DATA

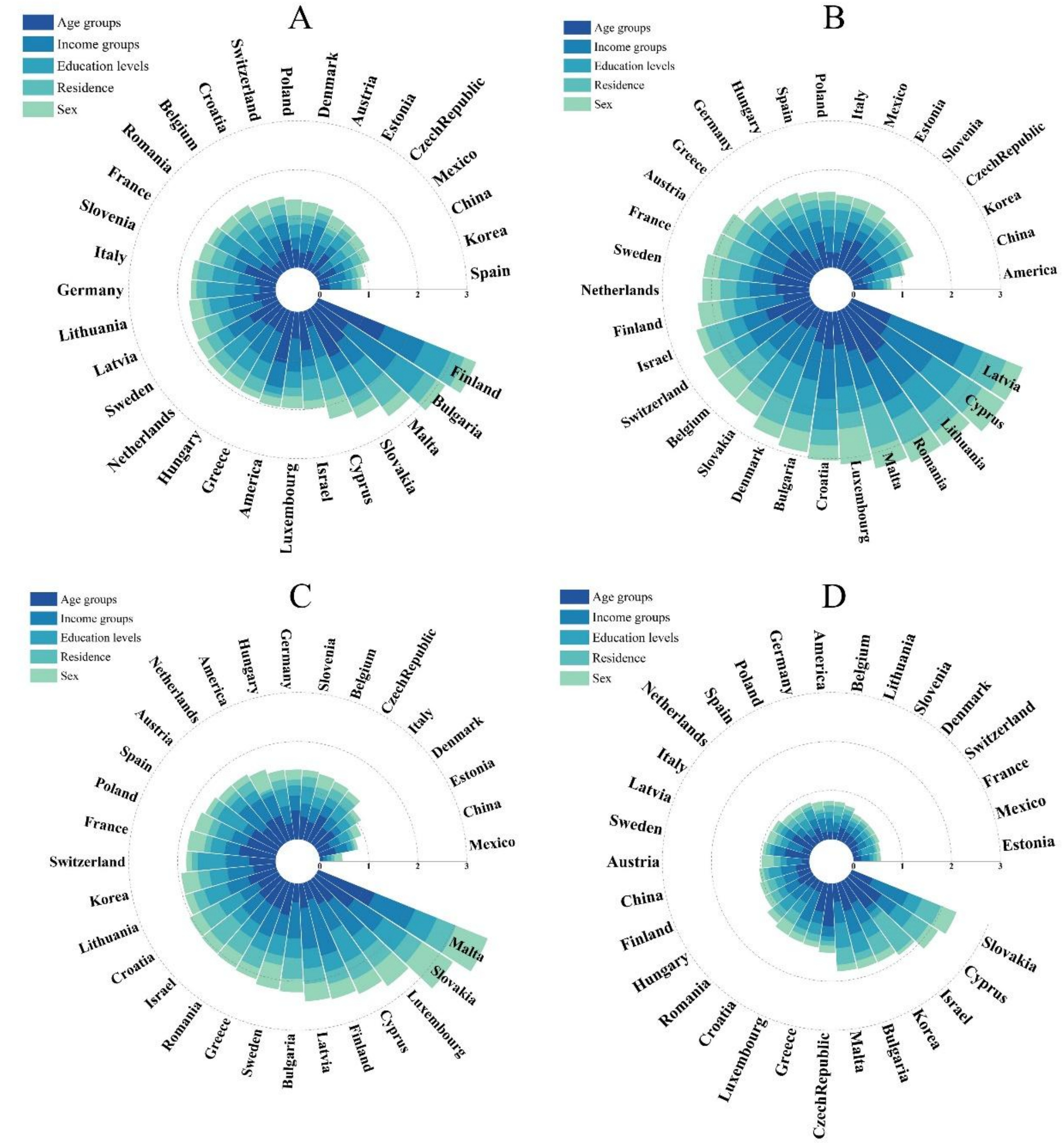

Supplementary Figure 1. Disease-specific relative inequality index for medication in older adults according to dimension. (A) Hypertension; (B) Diabetes; (C) Heart diseases; (D) Lung diseases.

© 2024. Wang M et al. Published online at http://www.aginganddisease.org/EN/10.14336/AD.2024.0626

SUPPLEMENTARY DATA

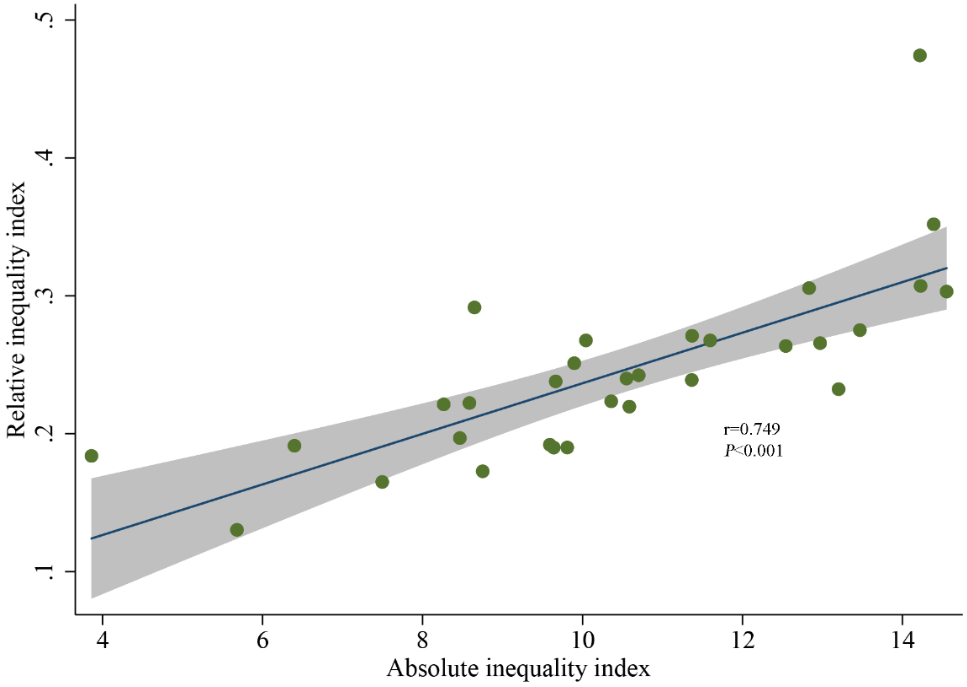

Supplementary Figure 2. Association between inequality index and UHC index for service capacity and access.

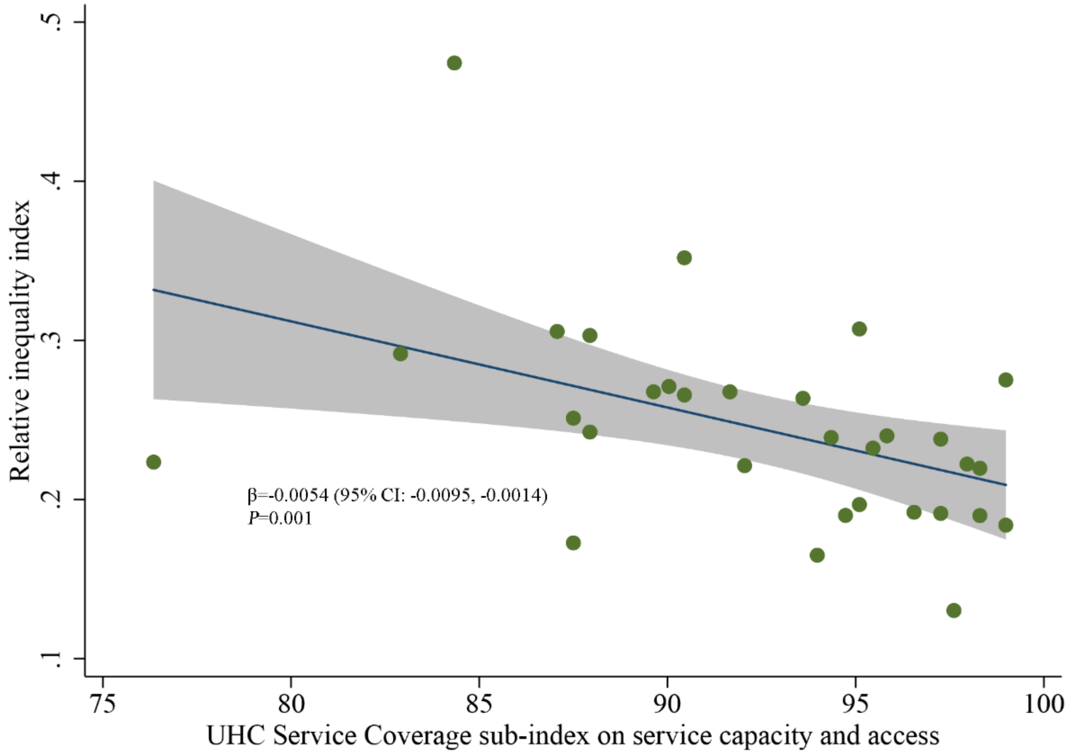

Supplementary Figure 3. Correlation between relative inequality index and absolute inequality index.
CI, confidence interval.

© 2024. Wang M et al. Published online at http://www.aginganddisease.org/EN/10.14336/AD.2024.0626

SUPPLEMENTARY DATA

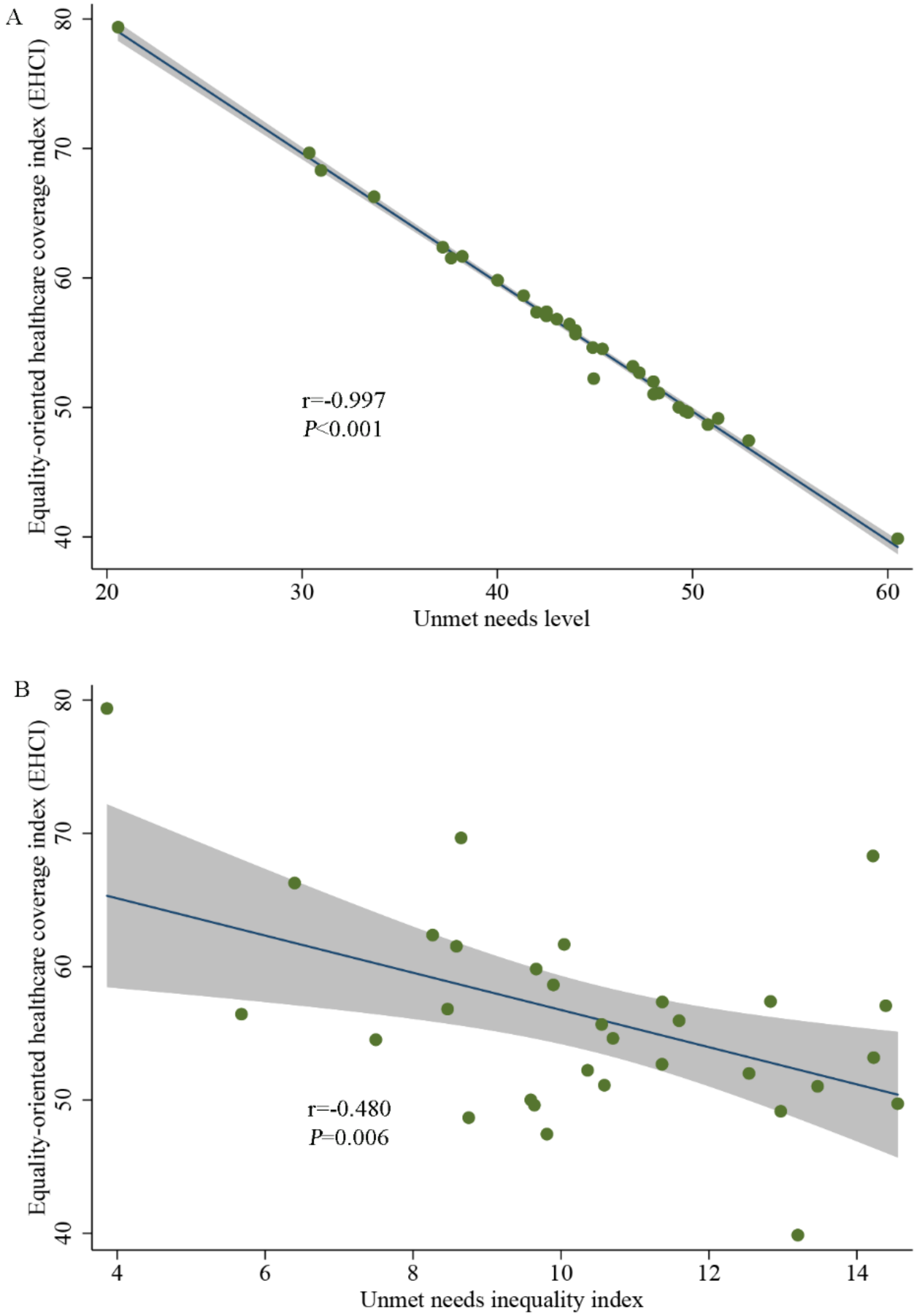

Supplementary Figure 4. Correlation between equality-oriented service coverage index (ESCI) in older adults with unmet needs level and inequality.

© 2024. Wang M et al. Published online at http://www.aginganddisease.org/EN/10.14336/AD.2024.0626
